# Doxycycline Inhibits Inflammation-Induced Lymphangiogenesis in Mouse Cornea by Multiple Mechanisms

**DOI:** 10.1371/journal.pone.0108931

**Published:** 2014-09-30

**Authors:** Longhui Han, Wenru Su, Jingwen Huang, Jingwen Zhou, Sujuan Qiu, Dan Liang

**Affiliations:** 1 State Key Laboratory of Ophthalmology, Zhongshan Ophthalmic Center, Sun Yat-sen University, Guangzhou, China; 2 Hebei Provincial Key Laboratory of Ophthalmology, Hebei Provincial Eye institute, Hebei Eye Hospital, Xingtai, China; Penn State University, United States of America

## Abstract

Lymphangiogenesis is significantly involved in the pathogenesis of diseases, including graft rejection, cancer metastasis and various inflammatory conditions. The inhibition of lymphangiogenesis has become a new therapeutic target for the treatment of these diseases. Here, we explored the anti-lymphangiogenic effects of doxycycline in inflammation-induced lymphangiogenesis (ILA) in the cornea and the underlying mechanisms. In the present study, mice with ILA of the cornea were treated with topical doxycycline (0.1%) or vehicle control. Lymphangiogenesis was quantified using corneal immunostaining of lymphatic vessel endothelial hyaluronan receptor-1 (LYVE-1). Human dermal lymphatic endothelial cells (HDLECs) and a murine macrophage cell line (RAW264.7) were used to further explore the underlying mechanisms of doxycycline-mediated anti-lymphangiogenesis *in vitro*. Our results showed that doxycycline treatment dramatically inhibited ILA in the mouse cornea (*p*<0.001), with a significant decrease in vascular endothelial growth factor (VEGF)-C/VEGF receptor 3 signalling, macrophage infiltration and inflammatory cytokine expression. Doxycycline also significantly inhibited VEGF-C-induced HDLEC proliferation *in vitro* by modulating the PI3K/Akt/endothelial nitric oxide (NO) synthase (eNOS) pathway and significantly suppressed interleukin-1β (IL-1β), TNF-α and VEGF-C production in the RAW264.7 cell line by modulating the PI3K/Akt/nuclear factor-kappaB (NF-κB) pathway. Additionally, doxycycline treatment dramatically reduced the phosphorylation of NF-κBp65, Akt and eNOS in ILA and significantly inhibited matrix metalloproteinases (MMPs) activity *in vitro* and in ILA. In conclusion, doxycycline inhibited ILA, possibly through suppression of VEGF-C signalling, macrophage function and MMPs activity. This observation suggests that doxycycline is a potential therapeutic agent for lymphangiogenesis-related diseases.

## Introduction

The lymphatic vascular system has multiple functions in normal physiology, including tissue fluid balance maintenance, immune surveillance, lipid absorption and inflammation resolution [1]. The formation of new lymphatic vessels (lymphangiogenesis) is also crucially involved in the pathogenesis of diseases, including graft rejection, cancer metastasis and various inflammatory conditions [1], [2], [3], [4], [5]. Therefore, inhibition of lymphangiogenesis has become a new therapeutic target for the treatment of these diseases [1], and currently, safe and effective methods of inhibiting lymphangiogenesis are being sought.

The cornea is an attractive system to investigate lymphangiogenesis because it is readily accessible for experimental manipulation and because the normal cornea is devoid of lymphatic vessels. Moreover, lymphangiogenesis underlies many corneal diseases that involve vision-threatening conditions, including corneal graft rejection, herpes simplex keratitis, chemical burns and quality-of-life-deteriorating diseases such as dry-eye syndrome [3], [6], [7], [8], [9], [10]. To search for novel therapeutic targets in lymphangiogenesis, we adopted a model of inflammation-induced lymphangiogenesis (ILA) in which the cornea is sutured.

Tetracyclines are a drug family that includes tetracycline, doxycycline, minocycline and other derivatives. Tetracyclines were originally developed as antibiotic agents, but these drugs have been discovered to possess striking variety of non-antibiotic properties. There are currently over 200 ongoing clinical trials of tetracyclines for the treatment of a wide range of diseases because of the drugs' multifunctional properties [11]. In particular, doxycycline is a long-acting, low-cost, semisynthetic tetracycline. Previous studies have also discovered that doxycycline can inhibit vascular endothelial growth factor (VEGF)-C signaling, macrophage function, matrix metalloproteinase (MMP) activity and inflammation [12], [13], [14], [15], [16], [17]. All of the above properties of doxycycline are closely related to anti-lymphangiogenesis, so we can infer that doxycycline can inhibit lymphangiogenesis. However, to our knowledge, no paper reporting that doxycycline can inhibit lymphangiogenesis has been published. Therefore, this study aimed to investigate the role of doxycycline in ILA in the cornea and its underlying mechanisms.

## Materials and Methods

### Ethics Statement and Animals

This study strictly adhered to the ARVO Statement for the Use of Animals in Ophthalmic and Vision Research and was approved and monitored by the Institutional Animal Care and Use Committee of Zhongshan Ophthalmic Center (Permit Number: SYXK (YUE) 2012-088). 135 female C57BL/6 mice (6–8 weeks, 19–22 g) were obtained from the Guangzhou Animal Testing Center, maintained under a 12-h light/dark cycle in a temperature- and humidity-controlled room and given ad libitum access to food and water, and the mice were studied in adherence with the ARRIVE guidelines. Additional enrichment and welfare were provided; for example, Animal health was monitored daily by the animal care staff and veterinary personnel. All surgery was performed under chloral hydrate solution anesthesia, and animals were kept warm during and after operation. All efforts were made to minimize suffering. The mice were sacrificed at the end of the 10-day experiment by uthanized in a carbon dioxide chamber filled with 100% CO_2_ for at least 10 min.

### Antibodies and Reagents

Doxycycline, hydroxypropyl-β-cyclodextrin, poloxamer 407, poloxamer 188, VEGF-C and lipopolysaccharides (LPS) were purchased from Sigma (St. Louis, MO, USA). Antibodies included, anti-LYVE-1, anti-VEGF receptor 3 (VEGFR3) (abcam, Hong Kong, China), anti-Akt, anti-phosphorylated Akt, anti-nuclear factor-kappaB (NF-κB) p65, anti-phosphorylated NF-κBp65, anti-IκB-α, anti-eNOS, anti-phosphorylated eNOS, anti-β-actin, horseradish peroxidase (HRP)-conjugated anti-mouse secondary antibody, HRP-conjugated anti-rabbit secondary antibody, Alexa Fluor 488-coupled goat anti-rat secondary antibody and Alexa Fluor 555-coupled goat anti-rabbit secondary antibody (Cell Signaling Technology, Inc., Danvers, MA).

### Preparation of Doxycycline Temperature-Sensitive Hydrogel (DTSH)

Topical 0.1% DTSH was prepared as described in our previous study [15]. In brief, doxycycline, hydroxypropyl-β-cyclodextrin, poloxamer 407 and poloxamer 188 were mixed at a mass ratio of 1∶24∶220∶35. The vehicle given to the control group contained all of the ingredients of the DTSH, except doxycycline.

### ILA Model and Treatment

**Figure 1:**
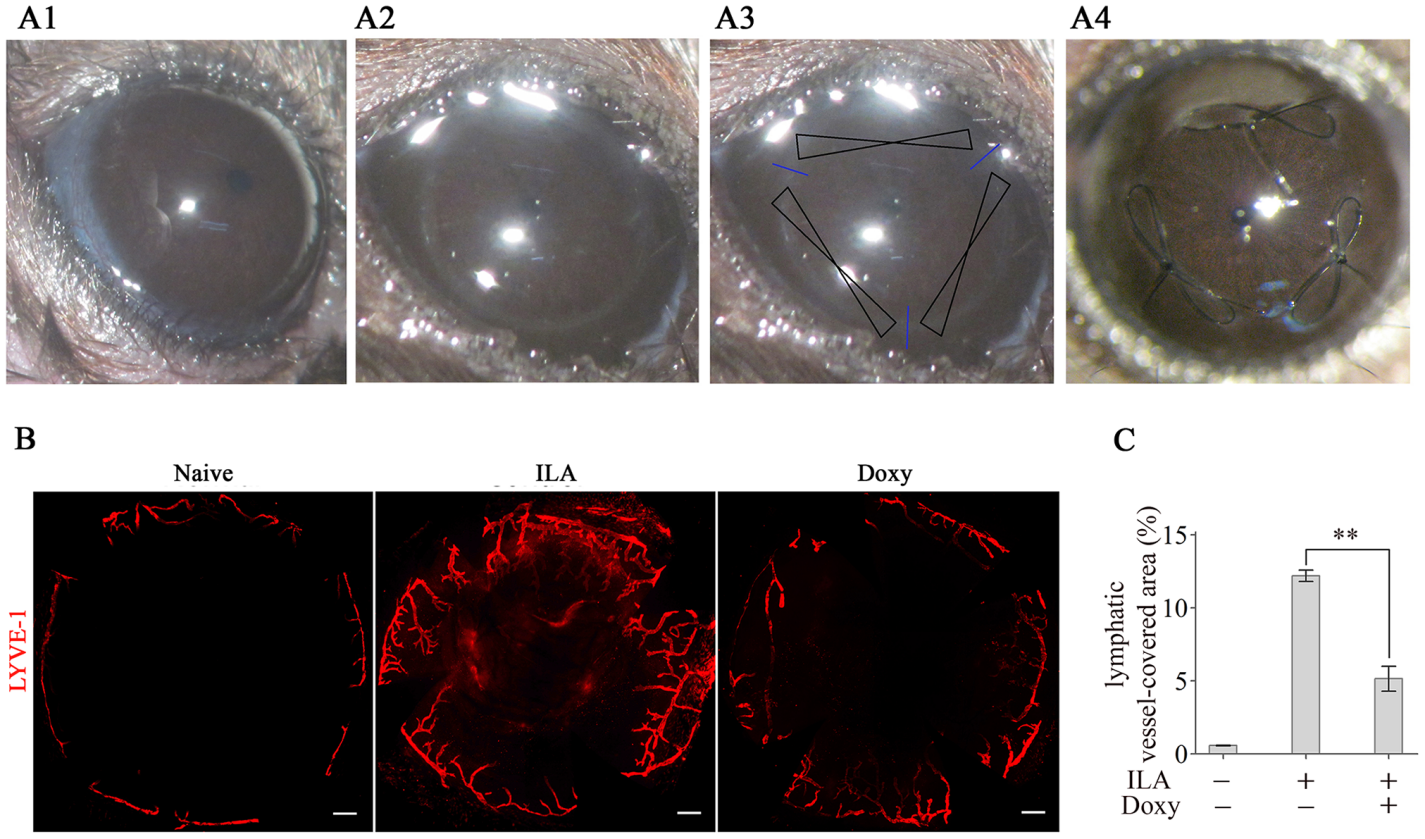
Doxycycline inhibits ILA in the cornea. (A): Animal model of ILA in the cornea. The four images represent a normal cornea (A1), a cornea marked with a circle (A2), a schematic diagram of the corneal sutures (A3) and post-operational corneal sutures (A4). (B): Representative images of LYVE-1-labelled corneal lymphangiogenesis in different groups. (C): Quantification of LYVE-1-labelled corneal lymphangiogenesis. **p<0.01. The scale bars represent 300 µm. Abbreviations: ILA, inflammation-induced lymphangiogenesis; Doxy, doxycycline; LYVE-1, lymphatic vessel endothelial hyaluronan receptor-1.

Each mouse was deeply anesthetised with an intraperitoneal injection of 10% chloral hydrate solution (0.04 mL/10 g) prior to surgery. A mouse model of ILA in the cornea (Fig. 1A) was used as described previously [18]. In brief, a 2-mm corneal trephine was gently placed on the centre of the cornea in the right eye of the anesthetised mice to mark a circle (Fig. 1A2) and to obtain a standardised model. Three 11-0 nylon sutures (Jiahe Inc., Taibei, Taiwan, China; NT0411) were placed intrastromally, with two stromal incursions each extending to approximately 120° of the corneal circumference (Fig. 1A3). The outer point of the suture placement was near the limbus, and the inner point was near the centre of the cornea (Fig. 1A4). The sutured mice were then randomly divided into a treatment group and a control group, and the sutures remained in place for the duration of the experiment (ten days).

There were 3 groups in the experiment: the treatment group (Doxy group), the control group and a naive group. Each group included 15 mice. The Doxy group and the control group were subjected to topical administration of 0.1% DTSH or vehicle, respectively (10 µl, 4 times/day). We began topical administration when the mice completely awakened post-suturing, and administration was continued for 10 days. In contrast, no treatment was given to the naive group. Three mice from each group were then used for whole-mount immunofluorescence, frozen-section immunofluorescence, Western blotting, MMPs activity assay, or PCR detection, respectively.

### Immunostaining and Quantification

For whole-mount corneal LYVE-1 immunostaining, at the end of the 10-day experiment, the mice were sacrificed, and their eyes were removed and fixed with 4% (wt/vol) paraformaldehyde at 4°C overnight. The eyes were then rinsed in phosphate-buffered saline (PBS), and whole-mount corneas were excised under a biomicroscope. The corneas were washed three additional times in PBS and blocked with 3% bovine serum albumin (BSA) in PBST (0.3% (vol/vol) Triton X-100 in PBS) for 2 hours, followed by staining overnight at 4°C with anti-LYVE-1 (1∶200). On day 2, the tissue was washed, blocked and stained with Alexa Fluor 555-coupled goat anti-rabbit antibody (1∶400) for 2 hours, as described previously [19]. Corneas flat mounted on microscope slides with Vectashield mounting medium were then examined with a fluorescent microscope, and images were taken with a 5× objective. The partial images were merged to obtain a composite image of the whole cornea using Leica Application Suite 4.1.0 software.

The merged image of the whole-mount cornea was imported into the Adobe Photoshop CC programme. The “Polygonal Lasso Tool” was first used to define the total area of the cornea (Fig. S1A). Next, “Record Measurements” in the “Measurement Log” window was selected to show the number of pixels (Fig. S1C). The area outside the cornea and the areas without lymphatic vessels in the central cornea were then filled with black colour. “Brightness/Contrast” and “Levels” were also adjusted to clarify the lymphatic vessels (Fig. S1B). Finally, the “Calculations…” tool was used to select the lymphatic-vessel covered areas (Fig. S1D) (“Selections” on the “Result” drop-down list), and the number of pixels was viewed in the “Measurement Log” window (Fig. S1C).

For frozen-section corneal immunostaining, at the end of the 10-day experiment, the mice were sacrificed, and their eyes were removed and embedded in OCT. The eyeballs were cut into 7 µm frozen sections, fixed with 4% (wt/vol) paraformaldehyde for 10 min, followed by procedure for antigen retrieval with Quick Antigen Retrieval Solution for Frozen Sections (Beyotime, Haimen, Jiangsu, China), and double stained with anti-CD11b (1∶400) and anti-F4/80 (1∶400), followed by the secondary antibody Alexa Fluor 555-coupled goat anti-rabbit and Alexa Fluor 488-coupled goat anti-rat (1∶400).

CD11b and F4/80 double stained fluorescent signals from the frozen corneal sections (40× objective, corneal area approximately 600 µm from the limbus) were imported into the Adobe Photoshop CC programme. The “Count Tool” was then used to determine the number of positive cells, and the “Polygonal Lasso Tool” was used to define the area of the cornea.

### Cell Culture

Human dermal lymphatic endothelial cells (HDLECs) were purchased from ScienCell (Carlsbad, CA) and maintained in endothelial cell basal medium-2 with growth supplements (EBM-2 MV). For the proliferation assay, HDLECs were pre-incubated with different doses of doxycycline (0 µM, 10 µM, 20 µM, 40 µM or 80 µM) for 1 hour, followed by incubation with or without VEGF-C (20 ng/mL). The cells were allowed to proliferate for 24 hours, and 100 µL of cells from each well was transferred to a new 96-well plate with 10 µL of Cell Counting Kit-8 solution (Dojindo Laboratories, Kumamoto, Japan). The absorbance at 450 nm was then measured using a microplate reader. Cell viability was also assessed before and after treatment using trypan blue exclusion and examination under a phase-contrast microscope.

HDLECs were cultured with VEGF-C for 24 hours following 1-hour pre-culture in the absence or presence of doxycycline (40 µM). The supernatants from the culture media were then collected for an NO assay, and the cells were collected for PI3K activity analysis, Western blotting and MMP activity assays.

The RAW264.7 murine macrophage cell line [20], [21] (Shanghai Cell Bank of Academia Sinica, Shanghai, China) was cultured in Dulbecco's modified Eagle's medium supplemented with 10% heat-inactivated foetal bovine serum (FBS) and 2 mM L-glutamine. For LPS activation, the cells were pre-incubated with doxycycline (40 µM) for 1 hour, followed by incubation with or without LPS (0.5 µg/mL) for 16 hours. Next, the supernatants from the culture media were collected for ELISA analysis, and the cells were collected for PI3K activity analysis, real-time PCR, Western blotting and MMP activity assays.

### Real-Time PCR

RAW264.7 cells and 3 corneas pooled from 3 mice from each experimental group were used for the real-time PCR analysis. The primer sequences, hybridation temperatures, number of cycles, and the length of the Real-time PCR products are described in the Table 1. Total RNA from the cell or tissue lysates was extracted with an RNeasy Mini Kit (Qiagen, Valencia, CA), and cDNA was generated using an Omniscript RT Kit (Qiagen). VEGF-C, interleukin-1β (IL-1β) and TNF-α mRNA expression was then quantified using ABsolute SYBR Green ROX mix (Thermo, Waltham, MA). The samples were run in triplicate, and the relative expression of VEGF-C, IL-1β and TNF-α was determined by normalising the expression of each target to β-actin using the 2^−ΔΔCt^ method.

**Table 1:** Sequences of Primers Used for Real-Time PCR Experiments.

Gene	Primer Sequence	Hybrid. Temperature	Cycles	Length
VEGF-C	Forward: 5′-TTTGCCAATCACACTTCCTGC-3′	57°C	30	160 bp
	Reverse: 5′- ACACTGTGGTAATGTTGCTGG-3′			
IL-1β	Forward: 5′-GCAACTGTTCCTGAACTCAACT-3′	57°C	28	89 bp
	Reverse: 5′- ATCTTTTGGGGTCCGTCAACT-3′			
TNF-α	Forward: 5′-CAGGCGGTGCCTATGTCTC-3′	58°C	28	89 bp
	Reverse: 5′- CGATCACCCCGAAGTTCAGTAG-3′			
β-actin	Forward: 5′-GGCTGTATTCCCCTCCATCG-3′	57°C	21	154 bp
	Reverse: 5′- CCAGTTGGTAACAATGCCATGT-3′			

### NO Assay and ELISA

The NO levels in supernatants from culture media were measured using the Griess reaction. In brief, 50 µL of each sample was mixed with 0.1% N-1-naphthylethylenediamine dihydrochloride and 1% sulphanilamide at room temperature for 10 minutes. The absorbance at 550 nm was then measured using a microplate reader.

PI3K activity in cultured cells was determined using a PI3-Kinase ELISA Kit Pico Assay (Echelon Biosciences Inc., Salt Lake, UT). In brief, combine 30 µL cell lysates (50 µg) and 30 µL of 10 µM PIP2 substrate, and incubate for 2.5 hours at 37°C. Incubate primary and secondary PIP3 detector for 1 hour and 30 minutes respectively. Read plate at 450 nm, and analyze data.

The concentrations of TNF-α and IL-1β in the culture medium of the cultured RAW264.7 cells were detected using ELISA (eBioscience, San Diego, CA). In brief, an ELISA plate was coated with 100 µL/well of capture antibody and incubated overnight at 4°C. Next, add 100 µL/well of supernatant to the appropriate wells and incubate at room temperature for 2 hours. Read plate at 450 nm, and analyze data.

### Western Blot Analysis

Cell lysates or lysates from 3 pooled mouse corneas (50–100 µg of total protein) were separated on a polyacrylamide-SDS gel and electroblotted onto a nitrocellulose membrane (Bio-Rad, Hercules, CA, USA). After blocking with TBS/5% nonfat dry milk, the membrane was incubated with antibodies against VEGFR3, Akt, p-Akt, NF-κBp65, p-NF-κBp65, IκB-α, eNOS, p-eNOS and β-actin followed by incubation with an HRP-conjugated secondary antibody. The signals were visualised using enhanced chemiluminescence detection (Pierce, Rockford, IL).

### MMPs Activity Assays

MMP-2 and MMP-9 activity was measured using a fluorogenic peptide substrate (R&D Systems) following the manufacturer's protocol. Briefly, the MMPs substrate was diluted in TCN buffer (50 mmol/L Tris-HCl, 150 mmol/L NaCl and 10 mmol/L CaCl_2_; pH 7.5) and added to supernatants from corneal or cell lysates (pre-activated by aminophenylmercuric acetate for 1 hour) before incubation at 37°C. After 30 minutes, total MMP activity was determined on a fluorometer (FLX 800 Microplate Fluorescence Reader; Bio-Tek Instruments, Winooski, VT).

### Statistical Analysis

All data are expressed as the mean ± SD from at least 3 independent experiments, and statistical analyses were performed with Student t test using SPSS software (16.0; SPSS, Chicago, Ill). Differences were considered statistically significant at a P value of less than 0.05.

## Results

### Doxycycline inhibits ILA in the cornea

No corneal epithelial defects, corneal ulcers, conjunctival necrosis, or other adverse complications related to topical doxycycline were observed in any sutured corneas throughout the experiment. Typical images of corneal lymphangiogenesis are shown in Figure 1B. Untreated sutured corneas showed significant lymphangiogenesis compared with normal control eyes at the 10-day endpoint, and doxycycline treatment significantly inhibited corneal lymphangiogenesis at the 10-day endpoint compared with no treatment (Fig. 1B, 1C).

### Doxycycline inhibits VEGF-C/VEGFR3 signalling, macrophage infiltration and inflammatory cytokine expression in ILA

**Figure 2:**
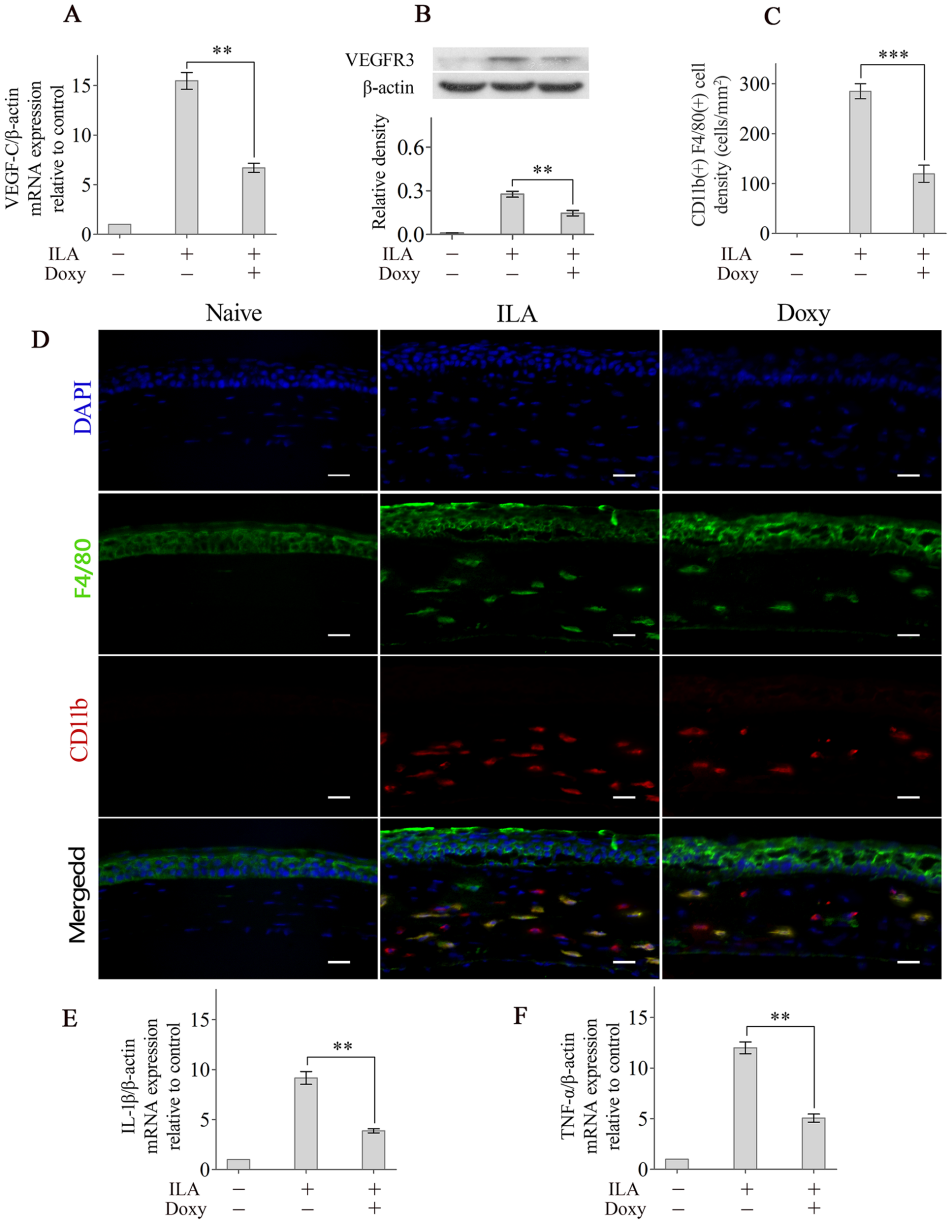
Doxycycline inhibits VEGF-C/VEGFR3 signalling, macrophage infiltration and inflammatory cytokine expression in corneas with ILA. The expression of VEGF-C mRNA (A) and VEGFR3 protein (B) in the corneas were detected using real-time PCR and Western blotting, respectively. (C): Quantification of CD11b and F4/80 labelled macrophages in the corneas. (D): Representative images of CD11b and F4/80 labelled macrophages in the corneas of different groups. The expression of IL-1β (E) and TNF-α (F) mRNA in the corneas was determined using real-time PCR. **p<0.01, *** *p*<0.001. The scale bars represent 25 µm.

Doxycycline treatment significantly reduced VEGF-C expression in corneas with ILA compared with expression in untreated corneas (Fig. 2A). Western blot analysis also showed that doxycycline significantly inhibited VEGFR3 expression (Fig. 2B). Additionally, the number of CD11b^+^ F4/80^+^ macrophages in corneas with ILA significantly increased compared with the number in naive corneas, and doxycycline treatment significantly decreased CD11b^+^ F4/80^+^ macrophages infiltration in the corneas with ILA (Fig. 2C, 2D). Real-time PCR analysis also showed that doxycycline treatment significantly reduced IL-β and TNF-α mRNA expression in corneas with ILA compared with expression in the untreated group (Fig. 2E, 2F).

### Doxycycline suppresses VEGF-C-induced HDLEC proliferation by modulating the PI3K/Akt/eNOS pathway

**Figure 3:**
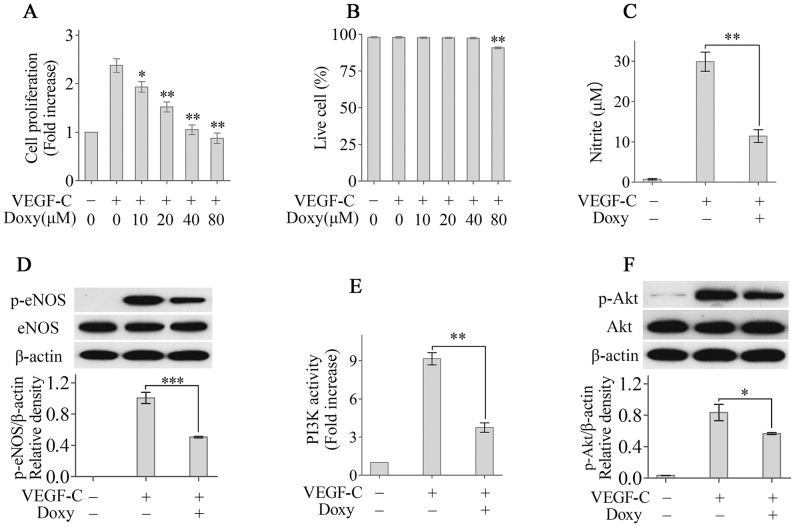
Doxycycline suppresses VEGF-C-induced HDLEC proliferation by modulating the PI3K/Akt/eNOS pathway. (A): Doxycycline inhibited the VEGF-C-induced proliferation of HDLECs in a dose-dependent manner. (B): The effect of different concentrations of doxycycline on cell viability. (C): The nitrite content in the supernatants was measured using the Griess reaction. (D): The expression of (phosphorylated) eNOS was determined using Western blotting. PI3K activity (E) and (phosphorylated) Akt (F) levels were determined using ELISA and Western blotting, respectively. *p<0.05, **p<0.01, ***p<0.001.

We first tested whether doxycycline could inhibit the proliferation of lymphatic endothelial cells (LECs) to explore the mechanisms of doxycycline-mediated suppression of lymphangiogenesis. Doxycycline inhibited VEGF-C-induced HDLEC proliferation in a dose-dependent manner (Figure 3A). In particular, VEGF-C-stimulated HDLEC proliferation was inhibited by 40 µM doxycycline to the level observed in unstimulated cultures, and this dose did not affect HDLEC viability (Figures 3A and 3B). Compared with 40 µM doxycycline, 80 µM doxycycline inhibited VEGF-C-stimulated HDLEC proliferation more effectively, but HDLEC viability was affected (Fig. 3A, 3B).

Endothelial eNOS-derived NO plays a critical role in lymphatic endothelial cell proliferation and lymphangiogenesis [22]. Therefore, we investigated whether doxycycline could modulate the phosphorylation of eNOS and NO production by HDLECs in order to explore the mechanism of doxycycline-mediated inhibition of HDLEC proliferation. Doxycycline treatment significantly reduced NO production by HDLECs stimulated with VEGF-C (Fig. 3C). Western blot analysis showed that doxycycline also significantly decreased eNOS phosphorylation (Fig. 3D). Previous studies showed that the PI3K/Akt pathway is critical for endothelial eNOS-derived NO release and VEGF-C induced lymphangiogenesis [23]. Therefore, PI3K activity and Akt phosphorylation were analysed using ELISA and Western blot, respectively, to further explore the mechanism of doxycycline-mediated inhibition of HDLEC proliferation. Our results showed that doxycycline treatment significantly reduced PI3K activity and Akt phosphorylation in HDLECs (Fig. 3E, 3F). These results implicated the PI3K/Akt/eNOS pathway in the doxycycline-mediated inhibition of HDLEC proliferation.

### Doxycycline suppresses LPS-induced macrophage activation by modulating the PI3K/Akt/NF-κB pathway

**Figure 4:**
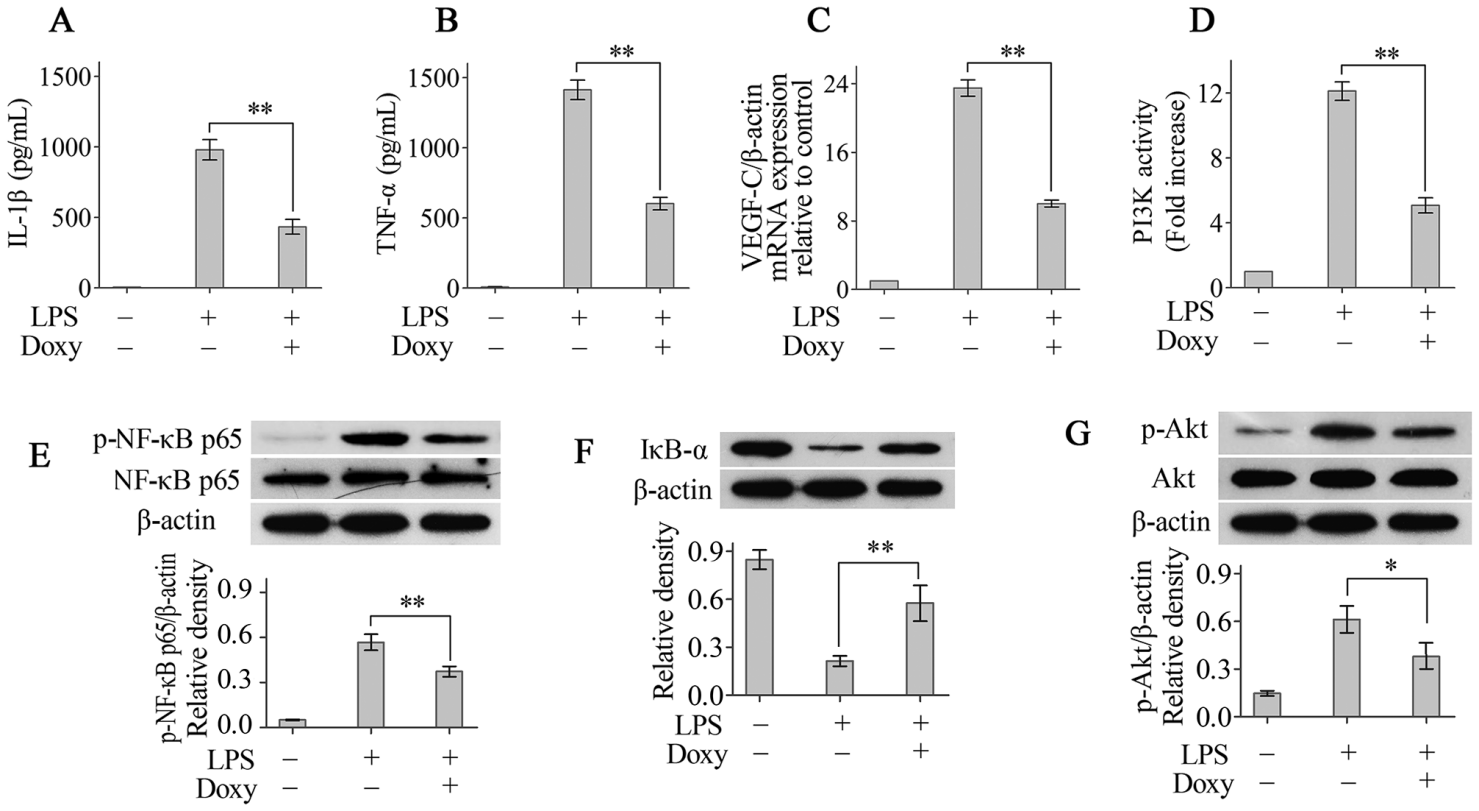
Doxycycline suppresses LPS-induced macrophage activation by modulating the PI3K/Akt/NF-κB pathway. The levels of IL-1β (A) and TNF-α (B) in the supernatants were measured using ELISA. The expression of VEGF-C mRNA (C) was determined using real-time PCR. Doxycycline treatment significantly reduced PI3K activity (D). The levels of (phosphorylated) NF-κBp65 (E), IκB-α (F) and (phosphorylated) Akt (G) were determined using Western blotting. *p<0.05, **p<0.01.

ELISA analysis showed that doxycycline treatment significantly inhibited IL-1β and TNF-α release by LPS-stimulated RAW264.7 cells (Fig. 4A, 4B). Additionally, real-time PCR showed that doxycycline significantly reduced the expression of VEGF-C mRNA in LPS-stimulated RAW264.7 cells (Figure 4C).

NF-κB is a heterodimeric transcription factor that plays a key role in inflammatory mediator release and macrophage activation. Therefore, we investigated whether doxycycline modulates NF-κB signalling in macrophages. Stimulation of RAW264.7 cells with LPS increased phosphorylated NF-κBp65 protein production, and doxycycline treatment significantly inhibited phosphorylated NF-κBp65 expression in RAW264.7 cells (Figure 4E). Most agents, including LPS, activate NF-κB through the degradation of IκB-α. IκB-α degradation exposes a nuclear localisation signal that leads to activation of NF-κB. Therefore, we also investigated whether doxycycline modulates NF-κB activity in RAW264.7 cells by inhibiting IκB-α degradation. As expected, doxycycline significantly inhibited IκB-α degradation in RAW264.7 cells (Figure 4F). Previous studies have shown that the PI3K/Akt pathway also plays an important role in NF-κB activation and macrophage function [23]. Therefore, PI3K activity and Akt phosphorylation were analysed using ELISA and Western blot, respectively, to further explore the mechanism of doxycycline-mediated inhibition of macrophage activation. Our results showed that doxycycline treatment significantly reduced PI3K activity (Fig. 4D) and Akt phosphorylation in RAW264.7 cells (Fig. 4G). Taken together, these results implicated the PI3K/Akt/NF-κB pathway in the doxycycline-mediated inhibition of macrophage activation.

### PI3K/Akt signalling is implicated in the doxycycline-mediated inhibition of ILA in corneas

**Figure 5:**
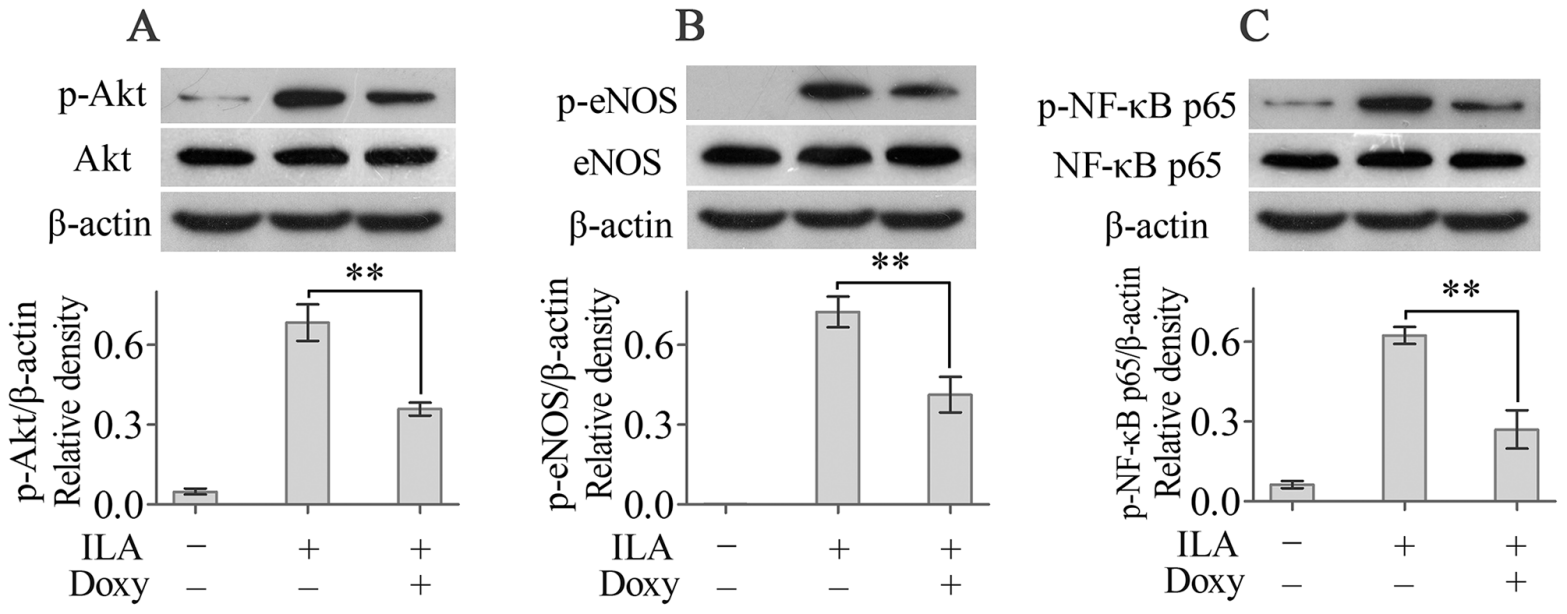
PI3K/Akt signalling is implicated in the doxycycline-mediated inhibition of ILA. The levels of (phosphorylated) Akt (A), (phosphorylated) eNOS (B) and (phosphorylated) NF-κBp65 (C) were determined using Western blotting. **p<0.01.

Our in vitro studies showed that PI3K/Akt signalling is involved in the doxycycline-mediated inhibition of HDLEC proliferation and macrophage activation. Therefore, we next asked whether the PI3K/Akt pathway was also involved in the doxycycline-mediated inhibition of ILA. Suture placement significantly increased the levels of phosphorylated Akt compared with the levels in the naive corneas, and doxycycline treatment significantly inhibited Akt phosphorylation compared with phosphorylation in the untreated sutured corneas (Fig. 5A). Doxycycline also significantly decreased the levels of phosphorylated eNOS and phosphorylated NF-κBp65 compared with the levels in the untreated corneas (Fig. 5B, 5C). Taken together, these results suggested that PI3K/Akt signalling is involved in the doxycycline-mediated inhibition of ILA.

### MMPs inhibition is involved in the doxycycline-mediated inhibition of ILA

**Figure 6:**
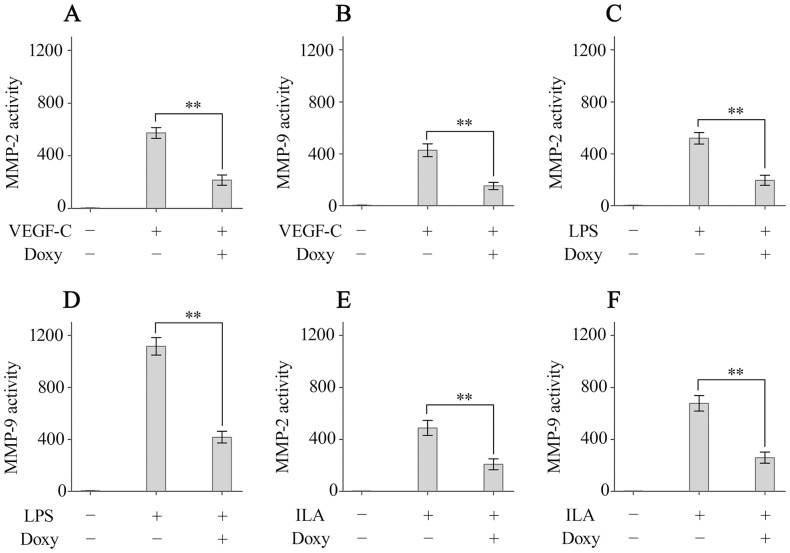
MMPs inhibition is involved in the doxycycline-mediated inhibition of ILA. (A, B): MMP-2 and MMP-9 activity in the supernatants of HDLEC lysates. (C, D): MMP-2 and MMP-9 activity in the supernatants of RAW264.7 cells lysates. (E, F): MMP-2 and MMP-9 activity in the lysates of corneas with ILA. **p<0.01.

MMPs, such as MMP2 and MMP9, play important roles in lymphangiogenesis [24], [25], [26], [27]. Doxycycline is a nonselective broad-spectrum inhibitor of MMPs that has been studied extensively and utilized in animal studies and clinical applications. Therefore, we next investigated whether the inhibition of MMPs is involved in the doxycycline-mediated inhibition of lymphangiogenesis. HDLECs were cultured with VEGF-C with or without doxycycline (40 µM) for 24 hours, and MMP-2 and MMP-9 activity was analysed in different experimental groups. Our results showed that doxycycline inhibited the activity of MMP-2 and MMP-9 produced by HDLECs (Fig. 6A, 6B). We also evaluated the inhibitory effects of doxycycline on the activity of MMPs produced by macrophages. As expected, treatment with doxycycline significantly inhibited MMP-2 and MMP-9 activity (Fig. 6C, 6D). Next, we analysed MMPs activity in ILA. Suture placement significantly increased MMP-2 and MMP-9 activity in ILA corneas compared to the normal control corneas, and doxycycline treatment significantly suppressed MMP-2 and MMP-9 activity compared to untreated control corneas (Fig. 6E, 6F). Taken together, these results indicated that the inhibition of MMPs is involved in the doxycycline-mediated inhibition of ILA.

## Discussion and Conclusion

Following the discovery of a striking variety of non-antibiotic properties of tetracyclines, the therapeutic applications of these drugs have been successfully extended [11]. Over the past few decades, tetracyclines have been successfully applied for the treatment of various diseases, including rheumatoid arthritis, periodontal disease, aortic aneurysm, stroke, shock, nasal polyps and metastatic tumours [11]. In the present study, we explored the role of doxycycline in ILA in the cornea and its underlying mechanisms. We found that doxycycline inhibited ILA in the cornea, with a significant decrease in VEGF-C/VEGFR3 signalling, macrophage infiltration and inflammatory cytokine expression. Doxycycline also inhibited VEGF-C-induced HDLEC proliferation and reduced pro-lymphangiogenic factor production by macrophages *in vitro*. Mechanistically, modulation of the PI3K/Akt pathway is implicated in the doxycycline-mediated inhibition of HDLEC proliferation, macrophage activation and ILA. In addition, the doxycycline-mediated inhibition of MMPs is involved in doxycycline-mediated ILA suppression. These findings provide the first evidence of the anti-lymphangiogenic properties of doxycycline.

Lymphangiogenesis differs greatly from angiogenesis in its function and molecular mechanism because of the specific functions of the lymphatic vessels in the maintenance of tissue fluid balance, immune surveillance, lipid absorption and inflammation resolution [1]. For example, lymphangiogenesis, but not angiogenesis, is responsible for graft rejection in organ transplantation [3]. Unlike angiogenesis, in which VEGF-A-VEGFR1/2 signalling plays a central role, VEGF-C-VEGFR3 signalling in lymphangiogenesis is the central regulator that controls lymphatic endothelial cell survival, proliferation and migration [1], [28]. Our *in vivo* results showed that doxycycline downregulated VEGF-C/VEGFR-3 signalling in ILA. *In vitro*, doxycycline treatment significantly reduced VEGF-C-induced HDLEC proliferation. Mechanistically, we found that the PI3K/Akt/eNOS-NO pathway was involved in the doxycycline-mediated inhibition of VEGF-C-induced HDLEC proliferation. This pathway is also involved in the doxycycline-mediated inhibition of ILA. These findings suggested that inhibition of VEGF-C signalling at least partially contributes to the doxycycline-mediated inhibition of lymphangiogenesis.

Macrophages are important cells in the innate immune response, and they play a critical role in lymphangiogenesis [29], [30], [31], [32], [33]. In particular, macrophages transdifferentiate into a lymphatic endothelial phenotype during lymphangiogenesis and are direct structural contributors to lymphatic endothelial walls [30], [33]. Furthermore, activated macrophages secrete pro-lymphangiogenic factors, such as VEGF-C and VEGF-D, which stimulate the division of pre-existing lymphatic endothelial cells or recruit more macrophages to inflamed or tumour tissues [34], [35], [36], [37]. In addition, the IL-1β and TNF-α produced by macrophages promote increased VEGF-C expression and recruit macrophages to inflamed or tumour tissues [38]. The present study showed that doxycycline inhibited macrophage recruitment and decreased VEGF-C, IL-1β and TNF-α expression in ILA. We also demonstrated that doxycycline significantly inhibited VEGF-C, IL-1β and TNF-α production in macrophages activated by LPS *in vitro*. Mechanistically, we found that doxycycline inhibited macrophage activation, possibly by modulating the PI3K/Akt/NF-κB pathway. Notably, we also observed that the PI3K/Akt/NF-κB pathway was implicated in the doxycycline-mediated inhibition of ILA. These findings suggested that doxycycline inhibits macrophage function, which may play an important role in the anti-lymphangiogenic properties of doxycycline.

MMPs are a family of zinc-dependent endopeptidases that are actively involved in lymphangiogenesis [24], [25], [26]. MMPs facilitate the lymphangiogenic factor-stimulated migration of lymphatic endothelial cells during lymphangiogenesis by disrupting cell-cell and cell-extracellular matrix interactions, which ultimately leads to the formation of new lymphatic vessels [24], [25], [26], [27]. Doxycycline is regarded as a nonselective, broad-spectrum inhibitor of MMPs [39], [40]. As expected, we showed that doxycycline significantly inhibited the MMPs activity induced by VEGF-C and LPS *in vitro*. Doxycycline treatment also significantly inhibited MMPs activity in ILA *in vivo*. These findings indicated that inhibition of MMPs is also involved in the doxycycline-mediated inhibition of ILA.

In conclusion, to our knowledge, this study is the first to demonstrate that doxycycline inhibits ILA. This inhibitory action may be attributable to the inhibition of VEGF-C signalling, macrophage function and MMPs activity. These findings confirm the anti-lymphangiogenic properties of doxycycline and support doxycycline as a potential therapeutic agent for the treatment of lymphangiogenesis-related diseases.

## Supporting Information

Figure S1 **The quatification of lyphatic vessel-covered areas.** (A): The “Polygonal Lasso Tool” was first used to define the total area of the cornea. (B): The area outside the cornea and the areas without lymphatic vessels in the central cornea were filled with black colour. “Brightness/Contrast” and “Levels” were adjusted to clarify the lymphatic vessels. (C): “Record Measurements” in the “Measurement Log” window was selected to show the number of pixels. (D): The “Calculations…” tool was used to select the lymphatic-vessel covered areas (“Selections” on the “Result” drop-down list).
(TIF)
