# Supplementary figures and images for: Doxycycline Inhibits Inflammation-Induced Lymphangiogenesis in Mouse Cornea by Multiple Mechanisms
Source: PLoS One. 2014 Sep 30;9(9):e108931. doi: 10.1371/journal.pone.0108931 (PMC4182529; doi:10.1371/journal.pone.0108931)

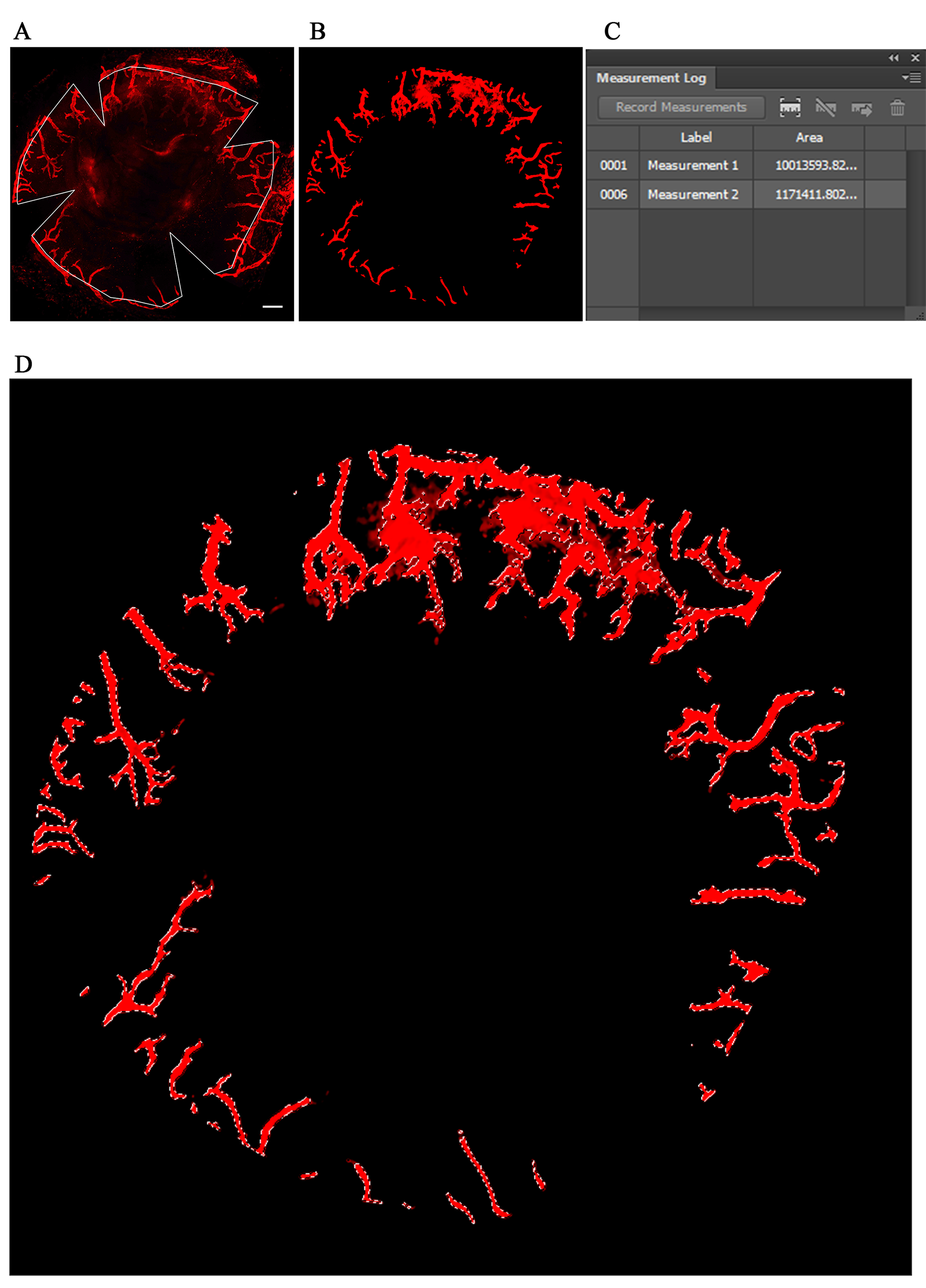

Supplement: Figure S1 — The quatification of lyphatic vessel-covered areas. (A): The “Polygonal Lasso Tool” was first used to define the total area of the cornea. (B): The area outside the cornea and the areas without lymphatic vessels in the central cornea were filled with black colour. “Brightness/Contrast” and “Levels” were adjusted to clarify the lymphatic vessels. (C): “Record Measurements” in the “Measurement Log” window was selected to show the number of pixels. (D): The “Calculations…” tool was used to select the lymphatic-vessel covered areas (“Selections” on the “Result” drop-down list). (TIF) [file pone.0108931.s001.tif]
